# Extracellular vesicle-based delivery to airway basal cells for durable gene therapy in cystic fibrosis

**DOI:** 10.3389/fbioe.2026.1855907

**Published:** 2026-07-15

**Authors:** Anastasiia Ivleva, Anna Demchenko, Svetlana Smirnikhina

**Affiliations:** Laboratory of Genome Editing, Research Centre for Medical Genetics, Moscow, Russia

**Keywords:** airway basal cells, cystic fibrosis, extracellular vesicles, gene therapy, peptide ligands, surface markers, targeted delivery

## Abstract

Airway basal cells are an appealing target for durable genetic therapy in cystic fibrosis because they are long-lived stem/progenitor cells that maintain and regenerate the pseudostratified airway epithelium. Correcting this compartment could, in principle, enable sustained restoration of CFTR function in differentiated epithelial lineages. However, selective *in vivo* targeting of basal cells remains challenging, owing to their position beneath the luminal epithelium and disease-associated remodeling of the cystic fibrosis airway. This Review examines the biological rationale for targeting airway basal cells in cystic fibrosis, with particular emphasis on the identification of surface markers that may permit selective delivery. We further survey peptide ligands with reported sequences for selected markers and consider extracellular vesicles as potential carriers for pulmonary delivery of genome-editing cargo to airway basal cells. Together, these considerations define the principal opportunities and current constraints of strategies aimed at targeting airway basal cells for durable cystic fibrosis therapy.

## 1 Introduction

Cystic fibrosis (CF) is a monogenic disease caused by pathogenic variants in the cystic fibrosis transmembrane conductance regulator (*CFTR*) gene. In the airway epithelium, loss of CFTR-dependent ion transport disrupts airway surface hydration, impairs mucociliary clearance, and promotes mucus retention, chronic infection, and persistent inflammation, thereby driving progressive lung disease (Matsui et al., 1998; Grasemann and Ratjen, 2023). Although highly effective CFTR modulators have substantially improved outcomes for many patients, these therapies do not correct the underlying mutation and do not fully address the needs of all people with CF, including those with variants that are not amenable to small-molecule modulator therapy, such as certain premature stop and splicing mutations (Middleton et al., 2019; Krishnamurthy et al., 2021). These limitations continue to motivate the development of durable etiologic strategies capable of restoring CFTR function directly within the airway epithelium.

In this context, airway basal cells (BCs) are an especially attractive therapeutic target. TP63^+^/KRT5^+^ BCs function as a major stem/progenitor population of the pseudostratified conducting airway epithelium, with the capacity for self-renewal and for generation of differentiated luminal cell types during homeostasis and repair (Rock et al., 2009; Walters et al., 2013). Correcting this compartment could therefore, in principle, provide more durable benefit than approaches limited to short-lived surface epithelial cells, as genetically corrected BCs should be able to generate functionally relevant descendants over time. Importantly, available threshold studies suggest that complete correction of the airway epithelium may not be required: substantial rescue of chloride transport and mucus transport has been observed when only a minority of airway epithelial cells are corrected, supporting the idea that even partial but appropriately targeted intervention may have therapeutic value (Johnson et al., 1992; Goldman et al., 1995; Zhang et al., 2009).

Despite this rationale, selective *in vivo* delivery to airway BCs remains technically challenging. BCs are positioned along the basement membrane beneath differentiated luminal cells in the pseudostratified airway epithelium, making them relatively inaccessible from the airway surface (Rock et al., 2010). For vectors delivered from the lumen, efficiency is further limited by mucus, mucociliary clearance, and epithelial tight junctions, which restrict access to basolateral membrane domains where entry receptors for certain airway gene-transfer systems are preferentially localized (Walters et al., 1999; Ferrari et al., 2002; Duncan et al., 2016; McCarron et al., 2023). In CF, these barriers are compounded by abnormal mucus properties and chronic inflammatory remodeling, emphasizing that successful BC targeting will require not only an effective cargo platform but also strategies that can penetrate airway barriers and engage accessible surface determinants on the appropriate epithelial subpopulation.

Extracellular vesicles (EVs) are attractive in this setting because they can encapsulate diverse therapeutic cargoes, including nucleic acids and proteins, while also offering opportunities for surface engineering to modulate cell targeting and uptake (Singh et al., 2020; Wang L. et al., 2023). In well-differentiated human airway epithelial models, EV-mediated delivery has already been used to transfer siRNA and protein cargo, and microvesicle-mediated CFTR protein transfer has been shown to restore chloride transport after transient airway surface preparation, supporting the relevance of vesicle-based systems for airway epithelial delivery (Singh et al., 2020). An EV-associated AAV6 platform has also been reported to enhance pulmonary gene transfer *in vivo* after intratracheal administration, suggesting that vesicle-based approaches may help overcome barriers that limit conventional airway delivery (Kwak et al., 2023). At the same time, their utility for BC targeting will depend not only on cargo loading and vesicle engineering, but also on the identification of surface ligands and receptors that can support selective and productive engagement of the target cell population.

In this Review, we examine the rationale for targeting airway BCs as a route toward durable etiologic therapy in CF, with particular emphasis on ligand-directed delivery strategies and EVs as adaptable carrier platforms. We first outline the biological role of airway BCs and the therapeutic logic for their correction, then discuss the anatomical and disease-specific barriers that complicate *in vivo* access to these cells. We next evaluate candidate surface markers for BC targeting and consider how their specificity, accessibility, and functional relevance may shape delivery design. Finally, we discuss the opportunities and current limitations of EVs and vesicle-mimetic systems for airway gene delivery, with a focus on how these platforms might be adapted for selective engagement of BCs.

## 2 Airway basal cells as a target population for durable etiologic therapy in cystic fibrosis

Airway BCs represent a promising cellular target for achieving long-lasting genetic correction in the conducting airways. These epithelial stem/progenitor cells can self-renew, persist through epithelial injury, and continuously generate differentiated luminal progeny during both homeostatic turnover and repair (Rock et al., 2009; Rock et al., 2010). This combination of durability and regenerative capacity makes BCs attractive anchors for therapeutic interventions aimed at sustained restoration of airway epithelial function. However, BCs reside on the basement membrane rather than the luminal surface, creating a practical delivery challenge: effective targeting often requires epithelial disruption, vector or administration strategies that overcome mucus and epithelial barrier, and/or receptor-targeted approaches that improve uptake by the desired cellular population (Duncan et al., 2016; Kim et al., 2016).

### 2.1 Airway basal cell identity

Airway BCs are epithelial stem/progenitor cells located on the basement membrane of the pseudostratified conducting airway epithelium. BCs are commonly defined by TP63 and KRT5/14 expression. Their stem and progenitor properties are well supported by mouse lineage-tracing studies demonstrating sustained contribution to epithelial maintenance and regeneration (Rock et al., 2009; Rock et al., 2011; Pardo-Saganta et al., 2015). In humans, evidence comes primarily from *ex vivo* differentiation, BC culture systems (including air-liquid interface models), and transcriptional reconstruction from single-cell datasets (Hackett et al., 2011; Ruiz García et al., 2019; Carraro et al., 2020).

#### 2.1.1 Localization

BCs are involved in the attachment of columnar epithelium to the basement membrane. They form desmosomes with adjacent columnar cells and are attached to the basement membrane via hemidesmosomes and other adhesion molecules (Evans et al., 1988). Their anatomical distribution differs substantially between mice and humans in ways that matter for therapeutic translation. In mice, BCs are enriched in the cartilage-containing proximal airway (trachea and main bronchi) and become scarce in distal intrapulmonary airways. In humans, BCs extend much further along intrapulmonary conducting airways and decline toward the respiratory bronchioles. This proximal-distal axis is relevant for translational work, because the airway territory in which BCs can be reached and replaced is broader in humans than in murine models (Rock et al., 2010).

#### 2.1.2 Differentiation pathways

BCs primarily give rise to secretory and multiciliated cells, and Notch signaling is a central regulator of this choice. In simplified terms, higher Notch activity biases differentiating progeny toward secretory programs, whereas reduced Notch activity permits multiciliogenesis (Rock et al., 2011; Gomi et al., 2015). Single-cell trajectory analyses of human mucociliary differentiation fit well with this framework: BCs first transition into intermediate “suprabasal” states (KRT13, KRT4) and then pass through club-like intermediates before diverging toward goblet (MUC5AC) and multiciliated (FOXJ1) lineages (Ruiz García et al., 2019). *In vivo* lineage-tracing combined with single-cell profiling in the murine trachea further indicates that BCs can also contribute to rarer epithelial types, including tuft cells, pulmonary neuroendocrine cells, and ionocytes (Montoro et al., 2018). Taken together, these data explain why BC correction is often discussed as a route to distribute therapeutic benefit across multiple downstream lineages over time (King et al., 2020; Plasschaert et al., 2024).

#### 2.1.3 Basal cell heterogeneity

Recent studies converge on a single idea: airway BCs are not a uniform stem-cell pool, but a plastic, environment-tuned ensemble of stem/progenitor states that collectively maintain and repair the epithelium. In human bronchial epithelium, single-cell cloning paired with transcriptomics reveals durable BC “variants” spanning multipotent, cycling, and secretory-leaning programs, and indicates that *ex vivo* conditions can shift how cells distribute along this continuum in a largely reversible manner (Reynolds et al., 2025). At the same time, this continuum contains experimentally tractable fractions: Cheng et al. identify a basal subset marked by the HLO1-6H5 antibody (which recognizes a glycosylated form of transferrin receptor, TFRC/CD71) and show that FACS-isolated HLO1-6H5+ BCs are enriched for clonogenic, bipotent stem/progenitor activity in bronchosphere assays (Cheng et al., 2024). *In vivo* injury models further underline BCs heterogeneity: Zou et al. show that BCs respond to TGF-β gradients, and perturbing canonical TGF-β/SMAD signaling changes proliferation, migration to damaged regions, and differentiation outcomes, thereby shifting repair toward hyperplasia and barrier defects (Zou et al., 2026). Taken together, these observations highlight the central role of BC heterogeneity in determining airway epithelial integrity and repair during injury and disease (Zhou et al., 2022).

### 2.2 Airway basal cells in cystic fibrosis: rationale, disease-associated alterations, and implications for targeting

**FIGURE 1:**
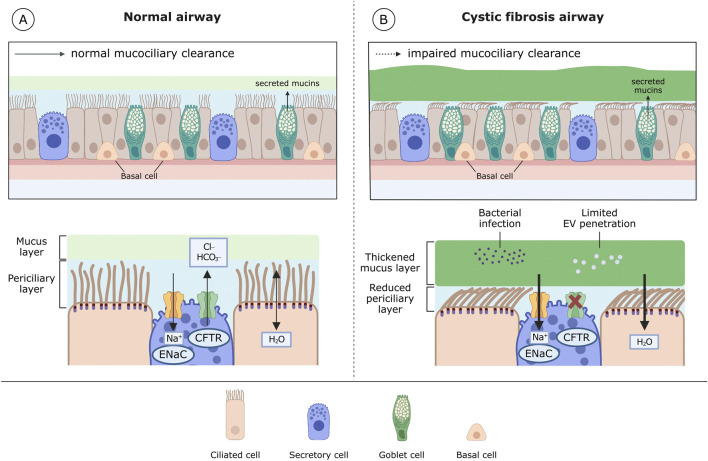
Structural and functional differences between normal and cystic fibrosis airways. In the normal airway **(A)**, CFTR-mediated chloride and bicarbonate secretion maintains airway surface hydration and supports effective mucociliary clearance. In cystic fibrosis **(B)**, loss of CFTR function causes airway surface dehydration, resulting in a thickened mucus layer and a reduced periciliary layer, which impair mucociliary clearance and promote chronic bacterial infection. This abnormal mucus environment constitutes a major physical barrier to inhaled therapeutic delivery vehicles, including extracellular vesicles. Created in https://BioRender.com.

Having established the normal biology of BCs, we now examine how CF alters this landscape and what these disease-associated changes mean for therapeutic delivery (Figures 1A,B).

#### 2.2.1 The rationale for basal cell-directed gene therapy

CFTR modulators improve outcomes in many patients, but they do not correct the causal *CFTR* mutation (King et al., 2020; Plasschaert et al., 2024). Some individuals carry *CFTR* variants that are not currently addressed by approved modulators, some show a lower-than-expected clinical response despite eligibility, and a subset experience adverse effects that can necessitate dose adjustment or discontinuation (Fajac and Sermet, 2021; Alicandro et al., 2024; Fajac et al., 2024). These limitations sustain interest in gene-based therapeutic strategies that aim to restore CFTR expression or function at the DNA or RNA level. Gene addition involves delivery of a functional CFTR transgene to airway epithelial cells, but efficient airway transduction, appropriate regulation of CFTR expression, and long-term therapeutic benefit remain difficult to achieve (Alton et al., 2015; Plasschaert et al., 2024). RNA-based approaches, including mRNA-based CFTR protein replacement and splice-modulating antisense oligonucleotides, can restore CFTR protein production or correct transcript processing without permanent genome modification, although their effects are transient or limited to specific *CFTR* variants (Michaels et al., 2020; Da Silva Sanchez et al., 2020; Allaire et al., 2023; Rowe et al., 2023). Genome editing aims to restore CFTR function by using programmable genome editing tools to modify the endogenous *CFTR* locus; however, its clinical translation requires controlled delivery of editing components and careful evaluation of unintended genomic effects (Maule et al., 2020; Allaire et al., 2023). Across these approaches, the choice of target cell population is one of the key determinants of therapeutic outcome.

BCs are attractive in this context because genetic correction in a self-renewing progenitor could be maintained long-term and propagated to differentiated progeny during epithelial turnover and repair (King et al., 2020; Vaidyanathan et al., 2020). Proof-of-concept genome-editing studies have demonstrated restoration of CFTR expression or function in airway-relevant CF models, including primary or immortalized airway epithelial cells and patient-derived airway basal cells differentiated at air-liquid interface (Maule et al., 2019; Suzuki et al., 2020; Vaidyanathan et al., 2020; Mention et al., 2023; Joynt et al., 2023; Bulcaen et al., 2024).

Beyond their progenitor capacity, BCs are also part of the CFTR-expressing landscape in human conducting airways. Single-cell RNA sequencing combined with functional measurements in human airway superficial epithelia indicates that secretory cells account for the largest fraction of total CFTR expression and CFTR-mediated anion transport, with BC clusters also contributing substantially (Okuda et al., 2021). Ionocytes exhibit very high per-cell CFTR expression but are rare (commonly reported at <1% of epithelial cells), limiting their contribution to tissue-level CFTR function despite their biological significance (Montoro et al., 2018; Plasschaert et al., 2018). This framing supports BC targeting not only for durability, but also because BC correction can, in principle, supply CFTR-restored luminal lineages over time (Vaidyanathan et al., 2020; King et al., 2020), aligning with the multi-lineage nature of CFTR expression across the airway epithelium. However, epithelial turnover is gradual, and corrected BCs do not immediately replenish the airway luminal compartment.

Building on this point, BC-directed correction should be considered complementary to targeting differentiated luminal cells. The conducting airway epithelium is a slowly renewing tissue under homeostatic conditions, and some mature luminal populations can persist for extended periods; for example, ciliated airway epithelial cells in mice have been reported to have half-lives of approximately 6 months in the trachea and 17 months in the lung (Rawlins and Hogan, 2008; Stripp and Reynolds, 2008; Watson et al., 2015). Even after injury, BC-mediated repair proceeds through sequential proliferation and redifferentiation rather than immediate epithelial replacement. In a chlorine-injury model, protein markers of ciliated and secretory cells reappeared mainly at days 7–10, and the regenerated epithelium still showed abnormalities at day 10 (Musah et al., 2012). Consequently, BC correction is expected to support durable epithelial repopulation, whereas delivery to differentiated CFTR-expressing luminal cells–particularly secretory cells, which contribute substantially to CFTR expression and anion transport–may be important for earlier restoration of ion transport and mucociliary function (Zhang et al., 2009; Okuda et al., 2021).

An important caveat is that the durability that makes BC-directed correction attractive also raises specific safety concerns. As edited BCs may persist and contribute to epithelial renewal, unintended editing outcomes could be maintained within the progenitor pool and transmitted to differentiated progeny. Although direct evidence in airway BCs remains limited, a recent study of CFTR cDNA insertion into the *CFTR* locus in airway BCs provides an example of functional and safety assessment: CFTR function was restored to non-CF levels, while only minor undesirable genomic changes were detected and edited stem cells retained differentiation potential (Vaidyanathan et al., 2024). At the same time, studies in human hematopoietic stem and progenitor cells indicate that editing of progenitor populations can be associated with undesired cellular and genomic effects: CRISPR-Cas9/AAV6-mediated HDR editing can induce senescence- and inflammation-associated programs (Conti et al., 2025), whereas base and prime editors can generate adverse transcriptional responses and unintended genomic alterations (Fiumara et al., 2024). These findings underscore the need for careful evaluation of both CFTR rescue and long-term effects on genome integrity and epithelial renewal in BC-directed genome editing.

The strategic framework of targeting airway progenitors for durable genetic correction extends beyond CF to other monogenic disorders affecting mucociliary function. Primary ciliary dyskinesia (PCD) provides a proof-of-concept example: lentiviral-mediated gene transfer of *DNAI1*, a PCD-associated outer dynein arm gene, restored ciliary beating in patient-derived airway epithelial cells (Chhin et al., 2009), and more recent work has demonstrated functional rescue of ciliary motility in patient-derived airway organoid models spanning multiple PCD genotypes (Huo et al., 2025). While the delivery constraints and target genes differ across diseases, the unifying principle remains that genetic correction of long-lived epithelial progenitors offers a route to sustained functional benefit across downstream lineages as the epithelium turns over and repairs itself.

#### 2.2.2 Cystic fibrosis remodels the basal compartment and creates delivery barriers

Chronic infection and inflammation in CF airways drive epithelial remodeling. Histopathologic studies and single-cell profiling of human CF airways report goblet cell hyperplasia, a decreased ciliated/goblet cell ratio, squamous metaplasia and increased thickness of reticular basement membrane (Carraro et al., 2021; Collin et al., 2021). However, reports on BC abundance in CF airways are inconsistent. Some studies describe BC expansion/hyperplasia during epithelial regeneration and remodeling (Hajj et al., 2007a; Adam et al., 2015), whereas others report a depletion in proliferating BCs (Carraro et al., 2021) or no difference relative to controls (Collin et al., 2021). These discrepancies likely arise from differences in airway region, disease stage, inflammatory context, and BC definitions, underscoring the need for further studies.

In addition to epithelial remodeling, CF imposes additional barrier by accumulation of thick, dehydrated mucus that impairs mucociliary clearance (Rubin, 2007; Figueira et al., 2022). Airway mucus is a viscoelastic mesh formed by polymeric, gel-forming mucins (notably MUC5AC and MUC5B) (Fahy and Dickey, 2010). In CF total mucin concentrations are increased by approximately fourfold compared with healthy controls, consistent with airway-surface liquid dehydration (Henderson et al., 2014; Batson et al., 2022). In parallel, chronic neutrophilic inflammation enriches mucus with extracellular DNA and filamentous actin released from degenerating neutrophils/NETs, which further increases sputum viscoelasticity and adhesivity (Tang et al., 2005). This abnormal mucus layer is a major obstacle for inhaled vector delivery in CF as it can physically trap gene delivery vehicles and reduce diffusion to the epithelial surface unless vectors are optimized for mucus penetration or preconditioning is done (Figure 1B) (Duncan et al., 2016; Kim et al., 2016; King et al., 2020).

#### 2.2.3 Therapeutic thresholds for CFTR rescue in the airway epithelium

Although efficient airway gene correction remains challenging, several lines of evidence suggest that restoring even modest CFTR activity can produce meaningful functional gains in the airway epithelium. In mixed bronchial epithelial cultures, approximately 10% non-CF cells were sufficient to functionally correct a primary culture of CF bronchial epithelial cells *in vitro* (Dannhoffer et al., 2009). Consistent with this, Farmen et al. reported that mixed airway epithelial cultures containing approximately 20% wild-type cells reached about 70% of the transepithelial chloride current observed in 100% non-CF cultures (Farmen et al., 2005). Ussing chamber analysis detected cAMP-dependent chloride transport with as little as 1% normal cells, whereas the less sensitive ^36^Cl efflux assay required at least 25%, indicating that apparent correction thresholds depend on the functional readout used (Illek et al., 2010). By contrast, when the endpoint is mucociliary function rather than ion transport alone, higher correction levels appear necessary: *CFTR* delivery to approximately 25% of surface epithelial cells restored mucus transport rates to those of non-CF airway epithelia *in vitro* (Zhang et al., 2009). Consistent with these threshold-type observations, recent gene-editing studies further support the concept that incomplete *CFTR* correction can yield biologically meaningful functional rescue (Sousa et al., 2024).

## 3 Candidate surface markers for targeted delivery to airway basal cells

Ligand-mediated targeted delivery refers to functionalizing a delivery vector with a targeting ligand (antibody, antibody fragment, peptide, aptamer, etc.) to promote selective binding to receptors on the intended cell population and thereby increase cellular uptake (Farokhzad et al., 2006; Rossin et al., 2008; Juliano, 2016; Tietjen et al., 2018; Fu et al., 2021). Functionally, improved delivery is attributed to stronger retention at the target cell surface, activation of receptor-mediated endocytosis, and reduced nonspecific uptake by off-target cells and tissues (Xu et al., 2013; Tietjen et al., 2018; Pearce and O’Reilly, 2019). In practice, efficiency of delivery depends on both target receptor accessibility in the relevant tissue compartment and extracellular barriers that limit vector transport to the cell surface (Pickles, 2004; Tietjen et al., 2018).

Several constraints are particularly important for delivering vectors to airway BCs in CF. BCs reside along the basement membrane within pseudostratified airway epithelium and are shielded from the airway lumen by differentiated luminal cells and tight junctions, which restrict apical-to-basal access (Shum et al., 2008; Rock et al., 2009; Ganesan et al., 2013). Moreover, many receptors that can support productive uptake are enriched at basolateral membranes of BCs, thereby further limiting receptor availability from the lumen (Walters et al., 1999; Pickles, 2004). In CF, thick, adherent mucus can slow or trap delivery systems before they reach the epithelial surface (Suk et al., 2009; Duncan et al., 2016; Kim et al., 2016; Morrison et al., 2019).

Barrier-mitigation strategies include mucolytic pretreatment, for example, N-acetylcysteine with or without recombinant DNase, which can improve penetration through CF sputum in relevant models (Suk et al., 2011). Another approach is transient epithelial barrier modulation with surfactant-like detergents such as lysophosphatidylcholine, which has been used to increase access to basolateral compartments and enhance gene transfer in airway models (Limberis et al., 2002; Cmielewski et al., 2010). In combination with these approaches, targeted delivery may reduce off-target uptake and improve internalization of therapeutic components once the vector reaches the appropriate membrane domain (Pickles, 2004; Tietjen et al., 2018).

In the following section, we evaluate candidate BC surface markers that could be used for receptor-targeted delivery, with emphasis on specificity, apical or basolateral accessibility in the airway epithelium, and the capacity to support productive internalization.

### 3.1 Criteria for selecting basal cell surface markers for targeted delivery

The selection of surface markers for targeted delivery to airway BCs should be based on a systematic, multi-parameter assessment that integrates biology of the target receptor with constraints imposed by the delivery route and tissue barriers. While no studies have explicitly defined criteria for BC targeting in CF, ligand-mediated drug delivery to other cell types studies have established key considerations that can be adapted to the BC context (Sahay et al., 2010; Muro, 2012; Rosenblum et al., 2018; Zhao et al., 2020).

*Specificity and selectivity.* The marker should be substantially enriched on the intended target cells and minimally present on off-target cell types (Glassman et al., 2020; Fu et al., 2022; Esapa et al., 2023). A large number of candidate targets show measurable expression across multiple cell types even when they are enriched in the target population, which can limit selectivity (Sahay et al., 2010; Kumar et al., 2016; Rosenblum et al., 2018). For airway BC targeting, candidate markers should show strong and preferential expression in BCs compared with luminal populations, including ciliated, secretory cells, and pulmonary ionocytes, to minimize off-target binding (Hackett et al., 2011; Plasschaert et al., 2018).

*Cell-surface localization and accessibility.* For luminal delivery, the basolateral membrane of BCs is difficult to access because BCs reside near the basement membrane and are shielded from the airway lumen by luminal cells and intact tight junctions (Walters et al., 1999; Pickles, 2004; Ganesan et al., 2013). Therefore, suitable markers should either be presented on the apical surface of BCs or be paired with strategies that enable access to the basolateral membrane (Limberis et al., 2002; Cmielewski et al., 2010; Thuenauer et al., 2017; McCarron et al., 2023).

*Sufficient surface expression.* A receptor should be present at appropriate density on the target cell surface and across a substantial fraction of the target population. Since low receptor abundance or high intercellular heterogeneity can limit binding, uptake, and effective delivery even when high-affinity ligands are used (Muro, 2012; Rosenblum et al., 2018; Woythe et al., 2021).

*Receptor internalization capacity.* For cargoes that require intracellular action, including mRNA therapeutics and genome editors, the receptor should support efficient receptor-mediated endocytosis after ligand binding, preferably through trafficking routes compatible with productive delivery rather than rapid lysosomal degradation (Sahay et al., 2010; Muro, 2012; Hammood et al., 2021).

*Low-risk receptor binding.* Binding of a vector to its target receptor should not trigger unintended receptor signaling or disrupt normal receptor function because these effects can reduce functional uptake and raise safety concerns (Muro, 2012; Saini et al., 2016; Esapa et al., 2023; Zou et al., 2021).

### 3.2 Candidate basal cell surface markers

A rational marker-selection strategy requires an integrated assessment across these criteria and an explicit understanding of the trade-offs involved. Highly specific markers may be present at low surface density, whereas receptors that internalize efficiently may not be accessible from the apical side. An additional challenge is that, for some candidate markers, key data are missing for one or more criteria, underscoring the need for further studies. Table 1 provides a systematic comparison of sixteen candidate BC markers with experimental evidence supporting their presence on the airway BC surface.

**TABLE 1:** Comprehensive evaluation of airway basal cell surface markers for targeted therapeutic delivery.

Priority	Marker (protein)	Gene/CD	Evidence in airway basal cells	Basal cell specificity	Stem enrichment evidence	Localization on basal cell surface	Internalization mechanism
High	NGFR (CD271, p75NTR)	NGFR/CD271	FACS: ITGA6^+^NGFR^+^ purification of human airway basal cells; Functional: bronchosphere formation from purified basal fraction (Rock et al., 2009). Flow cytometry: NGFR included in human bronchial epithelial subset panels for basal-cell identification (Bonser et al., 2021)	Flow cytometry: NGFR used to discriminate basal from secretory and ciliated airway epithelial populations (Bonser et al., 2021)	Bronchosphere-forming basal fraction defined as ITGA6^+^NGFR^+^ (Rock et al., 2009). NGFR abundance correlates with bronchosphere colony-forming efficiency during HBEC expansion (Lee et al., 2020)	Not reported	Internalization reported: clathrin-dependent endocytosis of ligand–p75 complexes to endosomal compartments (Bronfman et al., 2003)
High	ITGA6 (CD49f)	ITGA6/CD49f	FACS: ITGA6^+^NGFR^+^ purification of human airway basal cells (Rock et al., 2009). Flow cytometry: ITGA6 used in human bronchial epithelial subset panels for basal-cell identification/sorting (Bonser et al., 2021). FACS: detected in uncultured primary hBECs and conditionally reprogrammed hBECs (Cheng et al., 2024)	Flow cytometry: ITGA6 used to define basal cells relative to ciliated/secretory populations in airway epithelial panels (Bonser et al., 2021)	Basal cells ITGA6^+^NGFR^+^ form bronchospheres (Rock et al., 2009). Mouse trachea: CD49fbright/Sca1^+^/ALDH^+^ basal cells show tissue-specific stem cell properties (Ghosh et al., 2011)	Basolateral, luminal access limited in intact pseudostratified epithelium (Sonnenberg et al., 1991)	Internalization reported: antibody-mediated internalization of α6β1 and α6β4 integrins (Gaietta et al., 1994)
High	BCAM (CD239, Lutheran glycoprotein)	BCAM/CD239 (LU)	IF (human sinonasal epithelium): BCAM membrane signal on KRT5^+^ basal cells. Flow cytometry (human): BCAM heterogeneity within EpCAM^lo^ NGFR^hi^ basal gate. Tissue imaging + flow cytometry (mouse trachea): BCAM^hi^ vs. BCAM^lo^ basal subsets defined (Wang L. et al., 2023)	BCAM is considered as a marker of a subpopulation of basal cells (BCAM^hi^ vs. BCAM^lo^ basal cells) (Wang X. et al., 2023)	Functional validation *ex vivo*: BCAM^hi^ basal cells show increased proliferative activity (↑Ki67), larger Ki67^+^/P63^+^ colonies and improved wound closure vs. BCAM^lo^. ALI differentiation: BCAM^hi^ supports long-term growth and multilineage differentiation (Wang L. et al., 2023)	Basolateral, luminal access limited in intact pseudostratified epithelium (Wang X. et al., 2023)	Internalization reported (Kikkawa et al., 2018)
Moderate	PDPN (Podoplanin)	PDPN	FACS: PDPN identified in primary human tracheal basal cell cultures (Van De Laar et al., 2014). IHC/IF: PDPN used as basal-compartment marker in mouse trachea (Pardo-Saganta et al., 2015). FACS: PDPN used for human airway basal cells isolation (Clarke et al., 2025)	Human proximal airway epithelial sorting: PDPN reported enriched on basal cells, PDPN^−^ fraction largely non-basal (Clarke et al., 2025)	PDPN^high^ vs. PDPN^low^ basal fractions show differential colony-forming (Clarke et al., 2025)	Not reported	Internalization reported: clathrin-dependent endocytosis (Carrasco-Ramírez et al., 2016)
Moderate	CD151 (Tspan24, PETA-3)	CD151 (TSPAN24)	FACS: CD151^+^F3^+^ isolation of human airway basal cells (Hajj et al., 2007a). IHC/IF: CD151 detected on >95% of purified human airway basal cells (Hackett et al., 2011)	According to the Human Protein Atlas^1^, CD151 expression is observed in several types of respiratory epithelial cells	The CD151^+^/F3^+^ basal fraction is characterized by telomerase activity and provides regeneration of the mucociliary epithelium (Hajj et al., 2007b)	Not reported	Internalization reported: YXXφ motif-dependent endocytosis (Liu et al., 2007)
Moderate	F3 (Tissue factor, CD142)	F3/CD142	FACS (human airway epithelium): CD151^+^F3^+^ sorting enriches basal cells (Hajj et al., 2007a). Flow cytometry (human tracheal basal cultures): F3 detected on basal cells (Van De Laar et al., 2014). IF/IHC (human fetal trachea): F3 detected in TP63^+^ basal cells (Miller et al., 2020)	FACS (human airway epithelium): F3^+^ basal fraction separated from F3^-^ columnar epithelial cells (Hajj et al., 2007a). Fetal trachea: EGFR and F3 described as surface markers co-expressed in TP63^+^ basal cells (Miller et al., 2020)	The CD151^+^/F3^+^ basal fraction is characterized by telomerase activity and provides regeneration of the mucociliary epithelium (Hajj et al., 2007b)	Not reported	Internalization reported: antibody-mediated internalization (Kantak et al., 2025)
Moderate	Fn14	TNFRSF12A/CD266	IF (human distal lung): TNFRSF12A co-localized within KRT5^+^ basal layer. FACS (human distal lung): TNFRSF12A^+^ subset within EPCAM^+^ITGA6^+^ITGB4^+^ basal gate (Salahudeen et al., 2020). Flow cytometry (fresh human bronchial epithelium): basal-subset identified by TNFRSF12A (Carraro et al., 2021)	Within the basal compartment: TNFRSF12A identifies a minor KRT5^+^/p63^+^ basal subset. Non-basal airway epithelial expression not specified (Salahudeen et al., 2020)	Organoid assay (human distal lung): TNFRSF12A^hi^ basal fraction shows increased KRT5^+^ organoid formation vs. TNFRSF12A^neg^. Differentiation: TNFRSF12A^hi^-derived organoids generate SCGB1A1^+^ club and AcTUB^+^ ciliated cells (Salahudeen et al., 2020)	Not reported	Internalization reported: antibody-mediated internalization (Alvarez de Cienfuegos et al., 2020)
Moderate	ITGB4 (CD104)	ITGB4/CD104	FACS: EPCAM^+^ITGA6^+^ITGB4^+^ isolation of human epithelial progenitor/basal fractions followed by organoid assays (Salahudeen et al., 2020). IHC/IF: α6β4 localized to the basal plasma membrane of basal cells in differentiated human tracheal epithelium (Coraux et al., 1998). Mouse injury model: ΔNp63^+^/Krt5^+^ remodeling epithelium co-expresses integrin α6β4 (Vaughan et al., 2015)	Mouse airway regeneration: ITGB4 detected in club-associated progenitor populations (Kathiriya et al., 2020)	Organoid formation from EPCAM^+^ITGA6^+^ITGB4^+^ progenitor/basal fractions (Salahudeen et al., 2020)	Basolateral, luminal access limited in intact pseudostratified epithelium (Sonnenberg et al., 1991)	Internalization reported: antibody-mediated internalization of α6β1 and α6β4 integrins (Gaietta et al., 1994)
Low	EGFR	EGFR	IF/IHC (human fetal trachea): EGFR protein detected in TP63^+^ basal cells. FACS (human fetal lung epithelium): EPCAM^+^ gating followed by EGFR^+^F3^+^ enriches TP63^+^ basal cells (Miller et al., 2020). Transcriptome: EGFR enriched in human airway basal cells compared to differentiated epithelium (Hackett et al., 2011)	IHC (adult airway tissue): predominant apical staining in ciliated cells, basal cells are weakly immunostained (Tyner et al., 2006). IHC (asthma airway): EGFR staining detected in goblet cells (Takeyama et al., 2001)	Not reported	Basolateral, luminal access limited in intact pseudostratified epithelium (He et al., 2002)	Internalization reported: ligand-/antibody-mediated endocytosis (Liao and Carpenter, 2009)
Low	RON	MST1R/CD136	Flow cytometry (human tracheal basal cultures): MST1R detected on basal cells (Van De Laar et al., 2014)	IHC/IF (human trachea): staining across tracheal epithelium including non-basal cells (Van De Laar et al., 2014). IHC (human bronchus): RON detected in the apical surface of ciliated epithelium (Takano et al., 2000). Functional airway epithelium: RON activation regulates ciliary beat frequency (Sakamoto et al., 1997)	Not reported	Not reported	Internalization reported: antibody-mediated internalization with lysosomal trafficking (Yao et al., 2019)
Low	ITGA3 (CD49c)	ITGA3/CD49c	ISH, IHC (normal human bronchus): ITGA3 detected in basal cells (Boelens et al., 2007)	IHC (normal human bronchus): epithelial staining reported beyond the basal compartment (Boelens et al., 2007)	Not reported	Basolateral, luminal access limited in intact pseudostratified epithelium (Sheppard, 1998)	Internalization reported (Winterwood et al., 2006)
Low	CD9	CD9	Flow cytometry: CD9 detected on primary human tracheal basal cell cultures (Van De Laar et al., 2014)	According to the Human Protein Atlas^2^, CD9 expression is observed in several types of respiratory epithelial cells	Not reported	Not reported	Internalization reported: antibody-mediated endocytosis of CD9^+^ extracellular vesicles (Santos et al., 2019)
Low	TROP2	TACSTD2	IHC/IF (human bronchus): Trop2 membrane staining co-localized with KRT5^+^ basal cells (Liu et al., 2016)	IHC: detected in airway epithelium and alveoli in human lung (Lenárt et al., 2022)	TROP2 overexpression/knockdown modulates proliferation of basal cells (Liu et al., 2016)	Not reported	Internalization reported: antibody-mediated internalization (Fenn and Kalinsky, 2019)
Low	EpCAM	EPCAM/CD326	Flow cytometry (human bronchial epithelial cultures): EpCAM used as a pan-epithelial gate, that subsequently defines basal cells using lineage markers (Bonser et al., 2021). Flow cytometry (human bronchial epithelial progenitor populations): quantification of NGFR^+^EpCAM^+^ fractions within EpCAM^+^ epithelium (Lee et al., 2020). Flow cytometry (human IPF airway): EPCAM^+^KRT5^+^ basal-like population reported (Jaeger et al., 2022)	Pan-epithelial marker (Bonser et al., 2021)	Not reported	Basolateral, luminal access limited in intact pseudostratified epithelium (Wu et al., 2013)	Internalization reported: antibody-mediated endocytosis with endo-lysosomal sequestration (Lund et al., 2014)
Low	ICAM-1	ICAM1/CD54	FACS (human nasal epithelium): ICAM1 and NGFR are used together to isolate epithelial progenitors with basal-like phenotype and clonogenicity (Bravo et al., 2013)	ICAM-1 expression reported in various airway epithelial cells (Chong et al., 2021)	Not reported	Not reported	Internalization reported: multivalent ICAM-1 engagement triggers CAM-mediated endocytosis (Papademetriou et al., 2013)
Low	CD44	CD44	IHC (human trachea): CD44 staining on basal cells of surface epithelium. FACS (human trachea): coexpression of CD44 and NGFR was seen in all airway BCs (Hegab et al., 2012). FACS (human tracheal basal cultures): basal compartment described as marked by CD44 (Van De Laar et al., 2014)	IHC (bronchial biopsy) reports CD44 staining in columnar cells (Peroni et al., 1996)	Not reported	Not reported	Internalization reported: clathrin-independent endocytosis (Hua et al., 1993)

Markers were ranked into three priority groups based on basal cell specificity and evidence of stem enrichment. High priority: quantitative specificity and direct progenitor enrichment evidence. Moderate priority: functional isolation data but limited specificity or incomplete evidence. Low priority: broad epithelial expression or combinatorial gating required, with uncertain progenitor enrichment. BC, basal cell; IF, immunofluorescence; IHC, immunohistochemistry; IPF, idiopathic pulmonary fibrosis; ISH, *in situ* hybridization; FACS, fluorescence-activated cell sorting; ALI, air–liquid interface; hBECs, human bronchial epithelial cells.

1 The Human Protein Atlas. CD151 protein expression summary. https://www.proteinatlas.org/ENSG00000177697-CD151. [Accessed 8 April 2026].

2 The Human Protein Atlas. CD9 protein expression summary. https://www.proteinatlas.org/ENSG00000010278-CD9. [Accessed 8 April 2026].

To prioritize surface markers for ligand-directed therapeutic delivery to airway BCs, we grouped candidates into three groups based on two criteria: specificity for TP63^+^/KRT5^+^ BCs relative to other airway epithelial populations, and evidence that the marker enriches for stem or progenitor function. Group 1 (High priority) includes markers supported by quantitative specificity data and direct functional evidence of progenitor enrichment. Group 2 (Moderate priority) includes markers used for functional isolation or linked to progenitor-like subsets, but with weaker quantitative specificity, incomplete trafficking data, or reliance on limited datasets. Group 3 (Low priority) includes broadly expressed epithelial markers or targets that typically require combinatorial gating to achieve BC enrichment, with absent or uncertain evidence for stem or progenitor enrichment.

Group 1 includes NGFR, ITGA6, and BCAM, which together represent the strongest current candidates for targeting the airway BC compartment. NGFR and ITGA6 are well-established markers used in combination to enrich and isolate human airway BCs, with the ITGA6^+^NGFR^+^ fraction showing clonogenic bronchosphere-forming and regenerative activity, and NGFR expression also correlating with colony-forming efficiency during HBEC expansion (Rock et al., 2009; Lee et al., 2020; Bonser et al., 2021). BCAM complements these broadly validated markers by identifying a progenitor-enriched basal subset associated with increased proliferation, improved wound closure, and sustained differentiation in air–liquid interface culture (Wang X. et al., 2023).
Group 2 includes PDPN, CD151, F3, TNFRSF12A/Fn14, and ITGB4, which are supported by prospective-isolation or functional studies but remain more context-dependent markers of the basal compartment. CD151 and F3 are supported by functional isolation data, since CD151^+^F3^+^ cells from adult human airway epithelium showed telomerase activity and regenerated a differentiated mucociliary epithelium; however, their utility appears to depend on combinatorial gating rather than on strong evidence that either marker alone provides specific BC enrichment (Hajj et al., 2007b). TNFRSF12A/Fn14 has been used to identify a progenitor-like subset within the basal compartment, whereas ITGB4 was included in epithelial progenitor or BC gating strategies rather than evaluated as a standalone marker for airway BC targeting (Salahudeen et al., 2020). PDPN has also been reported in association with airway BCs, although evidence for BC specificity and progenitor enrichment remains limited and currently relies in part on recent preprint data (Van De Laar et al., 2014; Clarke et al., 2025).
Group 3 includes markers that are detected in airway BCs, but lack sufficient BC specificity and direct evidence of stem or progenitor enrichment to support selective targeting of the BC compartment. Within this group, some markers may still be relevant in the context of CF and airway inflammation. Although EGFR is detected in TP63^+^ fetal airway BCs, in adult airways it is more closely associated with epithelial activation and mucous remodeling, including enhanced EGFR-related signaling in CF (Takeyama et al., 2001; Stolarczyk et al., 2018; Miller et al., 2020). ICAM1 may be similarly relevant in inflamed or CF airways, as it can be induced on airway epithelium and has also been used together with NGFR to isolate epithelial progenitors (Hubeau et al., 2002; Bravo et al., 2013; Chong et al., 2021). The remaining Group 3 markers are heterogeneous but, in most cases, either show broader epithelial distribution, depend on combinatorial use for enrichment, or lack direct evidence for basal stem/progenitor selectivity. Accordingly, they may still be useful in multi-ligand or disease-context targeting strategies, but not as standalone markers for selective BC targeting.

These classifications indicate that marker performance is context-dependent and that no single receptor is likely to satisfy specificity, accessibility, and uptake requirements across models and disease states. Accordingly, experimental planning should evaluate a broad set of candidate markers and marker combinations together with airway delivery constraints. These considerations also favor delivery platforms that can be modularly functionalized with targeting ligands and engineered to navigate airway barriers, including EV-based systems.

## 4 Extracellular vesicles as delivery platforms for targeting airway basal cells

### 4.1 General characteristics of extracellular vesicles

EVs are a heterogeneous population of cell-derived particles delimited by a lipid bilayer and incapable of autonomous replication. They are widely recognized as important mediators of intercellular communication through the transfer of proteins, lipids, and nucleic acids. Traditionally, EVs have been classified according to their presumed biogenesis into exosomes, derived from the endosomal pathway, and microvesicles, formed by outward budding of the plasma membrane. However, because currently available isolation and characterization approaches do not generally permit unambiguous assignment of vesicles to a defined subcellular origin, the Minimal Information for Studies of Extracellular Vesicles 2023 (MISEV 2023) recommends use of the generic term ‘extracellular vesicle’ unless biogenesis has been rigorously demonstrated. Current guidelines also recommend the operational terms small EVs (<200 nm) and large EVs (>200 nm), with further classification based on physical characteristics, molecular composition, or cell source. MISEV2023 further distinguishes native EVs from EV mimetics, defined as EV-like particles produced through direct artificial manipulation. Although these particles may reproduce certain structural and membrane-associated features of native EVs, they are produced artificially rather than through physiological vesicle biogenesis (Welsh et al., 2024).

Native EV heterogeneity reflects at least two principal routes of vesicle formation. One major route involves inward budding of the endosomal membrane, generating intraluminal vesicles within multivesicular bodies, which are subsequently released into the extracellular space upon fusion of these compartments with the plasma membrane (Ostrowski et al., 2010; Baietti et al., 2012). Other EV populations arise through direct budding from the plasma membrane (Muralidharan-Chari et al., 2009). Together, these pathways give rise to differences in EV size, membrane composition, and surface characteristics. In experimental settings, native EVs are typically isolated from conditioned media or biological fluids after removal of cells and debris, followed by enrichment or separation by differential ultracentrifugation, density-gradient centrifugation, size-exclusion chromatography, ultrafiltration (including tangential flow filtration), polymer-based precipitation, or immunoaffinity capture (Tauro et al., 2012; Böing et al., 2014; Busatto et al., 2018). By contrast, EV mimetics are generated artificially–most commonly by serial extrusion or related mechanical disruption approaches–to produce cell-derived vesicles that retain selected membrane-associated features of the parental cells while providing substantially higher yields than naturally secreted EVs. Native EVs are therefore generally regarded as more physiologically relevant, whereas EV mimetics offer practical advantages in terms of yield and scalability, though they may not fully recapitulate the molecular composition or biological behavior of native vesicles (Jang et al., 2013; Wen et al., 2022).

**FIGURE 2:**
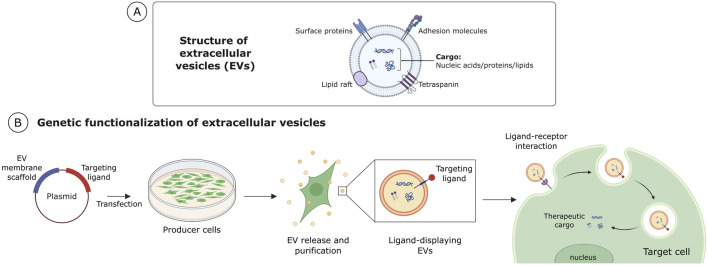
Extracellular vesicle structure and genetic functionalization for targeted delivery. **(A)** EVs are lipid bilayer-enclosed structures carrying diverse cargo, including nucleic acids, proteins, and lipids. Their membranes contain surface proteins, adhesion molecules, tetraspanins, and lipid raft-associated domains. **(B)** In genetic engineering approaches, producer cells are transfected with constructs encoding an EV membrane scaffold fused to a targeting ligand. The released EVs thereby display the ligand on their surface, enabling ligand–receptor interactions at the target cell and facilitating cellular uptake of therapeutic cargo. Post-isolation chemical surface-engineering strategies are illustrated in dedicated reviews (Richter et al., 2021; Johnson et al., 2023). Created in https://BioRender.com.

EVs are characterized by a complex molecular composition that reflects both their cellular origin and the pathway of vesicle formation. EV membranes are enriched in characteristic membrane-associated proteins, including the tetraspanins CD9, CD63, and CD81, as well as proteins involved in vesicle trafficking and membrane organization, such as ALIX, TSG101, flotillins, annexins, integrins, and heat shock proteins. In addition, EV membranes are enriched in specific lipids–notably cholesterol, sphingomyelin, phosphatidylserine, ceramides, and other sphingolipids–which contribute to membrane stability, vesicle formation, and interactions with recipient cells (Doyle and Wang, 2019; Gurung et al., 2021; Ghadami and Dellinger, 2023) (Figure 2A). Proteomic and lipidomic analyses further indicate that EV composition is not uniform, but varies across vesicle subpopulations and donor cell types, underscoring the molecular heterogeneity of EV preparations (Haraszti et al., 2016). Collectively, these compositional features are directly relevant to the use of EVs as delivery platforms, as they influence vesicle stability, interactions with recipient cells, and the efficiency of cargo protection and transfer.

### 4.2 Extracellular vesicles as therapeutic delivery platforms

Therapeutic delivery platforms are broadly divided into viral and non-viral systems. Viral vectors remain attractive because they can mediate efficient gene transfer, including in the lung, but their broader use is limited by vector-associated immune responses, safety concerns, and, in some cases, limited packaging capacity. Consequently, non-viral systems such as liposomes, lipid nanoparticles, polymeric nanoparticles, and EVs are being actively developed (Devaney et al., 2011; Shirley et al., 2020; Baliga and Dean, 2021; Bulcha et al., 2021; Bader et al., 2024). Among these, EVs are of particular interest as cell-derived, non-replicative carriers: they can protect cargo from degradation, exhibit relatively low immunogenicity and favorable biocompatibility, traverse biological barriers, and allow surface engineering for targeted delivery. Unlike liposomes and lipid nanoparticles, they also retain a native membrane composition, including endogenous proteins and lipids, that may facilitate interactions with recipient cells (Alvarez-Erviti et al., 2011; Vader et al., 2016; Zhu et al., 2017; Bader et al., 2024). EVs have accordingly been investigated for pulmonary administration, including intratracheal and nebulized delivery in preclinical models and an early clinical study (Porzionato et al., 2019; Shi et al., 2021). Their wider clinical translation, however, remains limited by vesicle heterogeneity, variable loading efficiency, and the lack of standardized large-scale manufacturing and characterization workflows (Vader et al., 2016; Bader et al., 2024).

### 4.3 Therapeutic cargo loading into extracellular vesicles

EVs can carry a broad range of therapeutically relevant cargo, including small-molecule drugs, oligonucleotides, mRNA, linear or plasmid DNA, proteins, and ribonucleoprotein complexes (Saari et al., 2015; Lamichhane et al., 2015; Yim et al., 2016; Gee et al., 2020; Han et al., 2021). For genome-editing applications, RNA and protein cargoes are often preferable for therapeutic use because transient nuclease activity may reduce the risk of off-target editing. This consideration is particularly relevant for preassembled ribonucleoprotein complexes, which deliver the nuclease and guide RNA as a functional complex without requiring prolonged intracellular expression (Kim et al., 2014; Ramakrishna et al., 2014; Lin et al., 2022). Consistent with this rationale, EV-based delivery platforms have been adapted for RNA- and RNP-based CRISPR cargoes, including CD9-HuR-mediated RNA loading, extracellular nanovesicle-mediated Cas9-sgRNA RNP delivery, and engineered EV-mediated enrichment of Cas9-or adenine base editor-containing RNP complexes (Li et al., 2019; Gee et al., 2020; Yao et al., 2021; Whitley et al., 2022). The practical use of these cargoes therefore depends on efficient loading into EVs.

Therapeutic cargo loading into EVs is generally classified as pre-loading during EV biogenesis and post-loading after EV isolation (Han et al., 2021). Pre-loading approaches include donor-cell incubation or transfection and engineered sorting systems that selectively enrich RNA or protein cargos into newly formed EVs. Two illustrative examples are CD9-HuR fusion constructs for RNA loading and the EXPLOR platform, which uses optically reversible protein-protein interactions to enrich cytosolic proteins into EVs (Yim et al., 2016; Li et al., 2019). Post-loading is performed on isolated EVs and includes passive incubation, electroporation, sonication, freeze-thaw cycling, and extrusion (Han et al., 2021; Kim et al., 2024). Pre-loading is generally better suited to complex biological cargos and better preserves EV integrity, whereas post-loading methods are simpler but may perturb vesicle structure; for example, electroporation can overestimate RNA loading because it induces siRNA aggregation (Kooijmans et al., 2013; Han et al., 2021). These approaches have been applied to genome-editing cargo, including Cas9/dCas9 mRNA, guide RNAs, and Cas9 ribonucleoprotein complexes. For such structurally complex cargos, pre-loading strategies are particularly suitable, although direct post-loading of Cas9 RNPs into purified EVs has also been demonstrated (Li et al., 2019; Yao et al., 2021; Majeau et al., 2022).

In CF, the EV cargos examined so far have been limited mainly to *CFTR* replacement and transcriptional regulation, rather than genome editing. EVs have been shown to transfer CFTR glycoprotein and its encoding mRNA to CF cells, with recovery of CFTR-dependent chloride transport (Vituret et al., 2016). Exosomes have been used to deliver siRNA into well-differentiated human airway epithelial cultures. In the same study, CFTR complementation in CF donor cells was achieved in a separate experiment through EV-mediated transfer of CFTR protein (Singh et al., 2020). Additional studies have used regulatory cargos to increase endogenous *CFTR* expression, including exosome-delivered BGas-targeting gapmers and exosome-packaged CFZF-VPR, a zinc-finger transcriptional activator of the *CFTR* locus (Villamizar et al., 2019; Villamizar et al., 2021). These approaches may enhance CFTR expression or function, but do not correct the causal mutation.

By contrast, EV delivery of nuclease-active Cas9, base editors, or prime editors for stable *CFTR* correction in airway BCs has not yet been reported in CF models. BC correction remains a central objective, because it could provide durable benefit through long-term renewal of corrected epithelial progeny. Support for this direction currently comes from non-CF lung studies: the safeEXO-CAS platform used ITGA6-engineered small EVs to deliver CRISPR/Cas9 components to lung epithelial cells, achieving editing frequencies of 7%–16% in mouse lungs after systemic administration and establishing a proof of concept for future EV-based genome-editing strategies in CF (Dubey et al., 2024).

### 4.4 Surface functionalization strategies for targeted delivery

Though EVs are promising drug delivery vehicles, unmodified EVs are limited by rapid clearance and low accumulation in target tissues and cells (Murphy et al., 2019). Their targeting ability can be enhanced by surface functionalization strategies, which are broadly divided into genetic and chemical engineering (Richter et al., 2021; Danilushkina et al., 2023).

Genetic engineering involves fusing the gene sequence of a targeting moiety to that of an EV membrane scaffold, such as LAMP2B, CD63, and other membrane-associated proteins. The resulting vector is then introduced into producer cells by transfection or transduction, leading to the generation of surface-modified EVs (Figure 2B). This approach enables predefined and relatively stable display of targeting ligands while largely preserving EV physicochemical properties. Once a stable producer line has been established, engineered EVs can also be harvested continuously without a separate post-isolation functionalization step (Salunkhe et al., 2020; Liang et al., 2021; Danilushkina et al., 2023). However, genetic engineering is time-consuming and costly, is largely restricted to genetically encodable ligands, and may affect EV biogenesis or composition. In addition, some fusion constructs are unstable; notably, LAMP2B-based fusions can undergo proteolytic degradation during exosome biogenesis (Hung and Leonard, 2015).

Alternatively, post-isolation chemical engineering enables the display of both natural and synthetic ligands without modifying producer cells. Covalent strategies include carbodiimide coupling (EDC/NHS), thiol-maleimide conjugation, and bioorthogonal click reactions such as SPAAC. A related strategy is metabolic glycoengineering, in which bioorthogonal groups are biosynthetically incorporated into membrane glycans of producer cells and subsequently displayed on EVs for click-chemical conjugation of targeting moieties (Rayamajhi and Aryal, 2020; Salunkhe et al., 2020; Danilushkina et al., 2023). By contrast, non-covalent approaches exploit electrostatic or hydrophobic interactions, as well as receptor-ligand binding. Despite this versatility, chemical approaches generally require post-modification purification and characterization and may alter EV physicochemical properties, thereby complicating reproducibility and clinical translation (Bahadorani et al., 2024). For CuAAC-based reactions, residual Cu(I) is a further concern because it can induce oxidative damage and is toxic to cells, making copper-free alternatives such as SPAAC preferable for biomedical applications (Hong et al., 2010).

Surface engineering can also be used to enhance fusogenic activity of vesicle-based delivery systems and thereby improve intracellular cargo release. This is particularly relevant for genome-editing cargoes, because internalized vesicles may remain trapped within endosomal compartments, limiting functional delivery (Ribovski et al., 2023; Berggreen et al., 2023). In this context, receptor recognition and membrane fusion represent complementary design elements. Targeting ligands promote selective binding to recipient cells, whereas fusogenic elements mediate fusion with the plasma membrane or with endosomal membranes after internalization, enabling cytosolic cargo release. Such fusogenic elements are commonly based on viral envelope glycoproteins or engineered variants with abolished receptor binding, although pH-sensitive fusogenic peptides have also been explored in non-viral delivery systems (Ahmad and Khan, 2022; Strebinger et al., 2023). This principle is illustrated by DIRECTED, a modular envelope-design platform that combines antibody-based targeting with separate viral fusogenic components and supports the use of fusogens from multiple viral families (Strebinger et al., 2023). Hamilton et al. applied a similar strategy to genome editing by combining scFv-based targeting with VSVGmut-mediated endosomal escape in Cas9-packaging enveloped delivery vehicles (Hamilton et al., 2024). Related EV engineering strategies have also combined high-affinity targeting domains with fusogenic glycoproteins to deliver biologics, including Cas9-sgRNA complexes, to primary human T cells (Stranford et al., 2024). Although these approaches have not yet been evaluated for airway BC targeting, they indicate that ligand-mediated targeted vesicle delivery may need to be coupled with enhanced fusogenic activity when the therapeutic cargo must reach an intracellular site of action.

### 4.5 Major classes of EV-targeting ligands

Several ligand classes have been used to actively retarget EVs, most commonly peptides, full antibodies, antibody fragments, and aptamers, each offering distinct targeting and engineering properties (Richter et al., 2021; Nan et al., 2022). Peptides are small, readily incorporated by either genetic display or post-isolation conjugation, although their utility may be constrained by limited proteolytic stability (Wang et al., 2022). Full antibodies provide very high affinity but are comparatively large, structurally complex, and less convenient for EV surface engineering (Nan et al., 2022; Koerselman et al., 2023). Antibody fragments such as Fab, scFv, and nanobodies preserve the antibody-based recognition principle while reducing molecular size and often improving engineering flexibility (Kooijmans et al., 2016; Pham et al., 2021; Koerselman et al., 2023; Alexander and Leong, 2024). Aptamers offer chemically synthesized, minimally immunogenic ligands, but commonly require stabilization against nuclease degradation for biomedical use (Zhou and Rossi, 2017; Ni et al., 2017). Ligand selection in EV design is therefore determined not only by target specificity and binding affinity, but also by practical considerations including molecular size, stability, production scalability, and compatibility with the chosen surface-functionalization strategy. As summarized in Table 2, these parameters are considered here for the major classes of EV-targeting ligands; surface-engineering strategies that increase vesicle fusogenicity are discussed separately in Section 4.4. Among the available targeting ligand classes, peptides appear particularly attractive for pulmonary EV engineering because of their structural simplicity, adaptability, and compatibility with both genetic and chemical display strategies. Accordingly, the following section focuses on candidate targeting peptides for airway BCs.

**TABLE 2:** Major classes of targeting ligands for EV surface engineering.

Characteristic	Peptides	Full antibodies	Antibody fragments (Fab/scFv/nanobodies)	Aptamers
Size	Small, ≤50 aa, ∼0,5–5 kDa (Sikorski et al., 2015; Henninot et al., 2018; Todaro et al., 2023)	Large, ∼145–150 kDa (Sikorski et al., 2015)	Intermediate. Fab - ∼50 kDa, scFv - ∼30 kDa, nanobodies - ∼15 kDa (Sikorski et al., 2015; Jin et al., 2023)	Very small, 25–50 nt, <20 kDa (Sikorski et al., 2015)
Affinity	Moderate-high, usually nM (pM for multivalent peptides) Newman and Benoit (2018), Todaro et al. (2023)	High, nM-pM (Freise and Wu, 2015)	High, nM-pM (Sikorski et al., 2015; Asaadi et al., 2021; Jin et al., 2023)	High; nM–pM (Jing and Bowser, 2011; Sikorski et al., 2015)
Stability	Limited, protease-sensitive, improved by modification (Lamers, 2022; Wang et al., 2022)	High, but formulation-dependent, susceptible to physical and chemical instability (Wang et al., 2007)	scFv low-moderate, Fab moderate, nanobodies high (Dumoulin et al., 2002; Röthlisberger et al., 2005; Alexander and Leong, 2024; Tadokoro et al., 2024)	Limited, nuclease-sensitive, improved by chemical modification (Kratschmer and Levy, 2017; Zhou and Rossi, 2017)
Production method	Chemical synthesis (SPPS) Sharma et al. (2023)	Mammalian expression (mainly CHO) Li et al. (2010), Zhang et al. (2022)	Recombinant expression (bacterial, yeast, mammalian) (Frenzel et al., 2013; Kang and Seong, 2020)	SELEX followed by chemical synthesis (Takahashi, 2018)
Functionalization compatibility	Genetic, chemical (Gabaran et al., 2025)	Chemical (Murphy et al., 2019; Danilushkina et al., 2023)	Genetic, chemical (Murphy et al., 2019; Danilushkina et al., 2023)	Chemical (Rayamajhi and Aryal, 2020; Danilushkina et al., 2023)
Immunogenicity risk	Low, sequence-dependent (Wang et al., 2022; Puig and Shubow, 2025)	Moderate. Reduced by humanization, but not eliminated (Hwang and Foote, 2005)	Generally lower than full antibodies, format- and humanization-dependent (Fernandes, 2018; Rossotti et al., 2022)	Very low, but sequence-dependent innate immune activation has been reported (Avci-Adali et al., 2013; Zhou and Rossi, 2017)

This table summarizes ligand classes used for EV targeting. Fusogenic ligands are discussed separately in Section 4.4.

### 4.6 Peptide ligands for basal cell surface markers

Based on the candidate BC surface markers listed in Table 1, we identified 25 peptide ligands with reported amino-acid sequences and compiled them for comparison in Table 3. To our knowledge, none of these ligands has been evaluated directly on airway BCs. As most were originally characterized in non-airway contexts, they should currently be regarded only as provisional candidates for EV-mediated delivery to this cell population. Their application to BC targeting will require systematic *de novo* validation, including assessment of binding specificity under physiologically relevant conditions, quantification of receptor-mediated internalization, and evaluation of potential functional consequences of receptor engagement. This issue warrants particular attention, because several receptors represented in Table 2, including integrins, EGFR and NGFR, are linked to epithelial adhesion, proliferation, and differentiation, respectively, and their engagement by an exogenous ligand cannot be assumed to be functionally inert (Tomellini et al., 2014; Zuo et al., 2017; Hsieh et al., 2023). At the same time, for many additional candidate BC markers listed in Table 1, no peptide ligands with defined amino-acid sequences are currently available, indicating that the repertoire of BC-directed targeting peptides remains incomplete and represents an important area for further investigation.

**TABLE 3:** Candidate peptide ligands for targeting basal cell-associated surface markers.

Target	Peptide name	Sequence	Genetically encodable	Internalization reported	Reported functional effects/liabilities	Model tested	References
ITGA6	RWY	CRWYDENAC	No	Yes	NR	Tumor imaging/nanodelivery, *in vitro* + *in vivo*	Xiao et al. (2019)
	S5	CRWYDANAC	No	Yes	NR	Pancreatic cancer imaging, *in vitro* + *in* *vivo*	Mei et al. (2020)
	4S5	RWYDANAGSGRWYDANAGSGRWYDANAGSGRWYDANA	Yes	Yes	NR	Melanoma imaging/therapy, *in vitro* + *in vivo*	Zhao et al. (2025)
	4S5NG	RWYDANAGSGRWYDANAGSGRWYDANAGSGRWYDANAGSGNNTHDLGVDVRLAGVQSVASSRRHKRFAGVGGSLAEALEAALEAALEAA	Yes	Yes	NR	Melanoma imaging/therapy, *in vitro* + *in vivo*	Zhao et al. (2025)
	P3	VSWFSRHRYSPFAVS	Yes	NR	NR	Cell adhesion/spreading assays with free peptide	Aina et al. (2007)
	AG-10	NRWHSIYITRFG	Yes	NR	NR	Cell adhesion/spreading assays with free peptide	Meng et al. (2010)
	AG-32	TWYKIAFQRNRK	Yes	NR	NR	Cell adhesion/spreading assays with free peptide	Meng et al. (2010)
	CYESIKVAVS	CYESIKVAVS	Yes	Yes	NR	Aerosol polyplexes to lung tumors, *in vivo*	Taschauer et al. (2019)
ITGA6/ITGB4	AG86	LGGLPSHYRARNI	Yes	NR	NR	α6β4-targeted liposomes, *in vitro*	Levine and Kokkoli (2017)
NGFR	VNLQNPY	VNLQNPY	Yes	NR	NR	p75NTR + cells	Kritz (2013)
	VYARSMN	VYARSMN	Yes	NR	NR	p75NTR + cells	Kritz (2013)
F3 (tissue factor/CD142)	TF-binding peptide	CGGGKFRVFALTR	Yes	Yes	Part of TF/coagulation axis	Vascular injury, *in vivo*	Levy et al. (2023)
Fn14 (TNFRSF12A)	D-FNB	d-CHPREVDDVELYSTVFGH	No	Yes	A signaling receptor (TWEAK/Fn14, NF-κB/MAPK), agonism by D-FNB not clearly reported	Liver fibrosis, *in vivo* + *in vitro*	Huang et al. (2017)
ITGA3	LXY30	cdG-Phe(3,5-diF)-G-Hyp-NcR	No	Yes	NR	Glioblastoma, NSCLC, breast cancer, *in vitro* + *in* *vivo*	Xiao et al. (2019)
	cNGQGEQc	cNGQGEQc	No	NR	NR	NSCLC, *in vitro* + *in vivo*	Zou et al. (2020)
​	GD-6	KQNCLSSRASFRGCVRNLRLSR	Yes	NR	NR	*in vitro* binding assays, free peptide	Gehlsen et al. (1992)
	TSP1-derived α3β1 site	FQGVLQNVRFVF	Yes	NR	Anti-angiogenic/adhesion-modulating motif	Angiogenesis, *in vitro* + *in vivo*, free peptide	Krutzsch et al. (1999)
	LABL	ITDGEATDSG	Yes	Yes	Blocks ICAM-1/LFA-1	Inflammation, *in vitro*	Khondee et al. (2011)
	cLABL	cyclo (1,12)PenITDGEATDSGC	No	Yes	Blocks ICAM-1/LFA-1	Inflammation, *in vitro*	Zhang et al. (2008)
	γ3	NNQKIVNLKEKVAQLEA	Yes	Yes	NR	Inflammation, *in vitro*	Garnacho and Muro (2017)
CD44	CD44BP/A5G27	RLVSYNGIIFFLK	Yes	Yes	Reported to inhibit FGF2-linked migration/invasion/angiogenesis	Tumor, *in vitro* + *in vivo*	Hibino et al. (2004)
EGFR	GE11	YHWYGYTPQNVI	Yes	Yes	Reported as much less mitogenic than EGF	EGFR + tumors, *in vitro* + *in vivo*	Li et al. (2005)
	D4 (L1)	LARLLT	Yes	Yes	NR	EGFR + tumors, *in vitro*	Song et al. (2009)
	EBP (CY12)	CMYIEALDKYAC	No	Yes	NR	EGFR + tumors imaging, *in vitro* + *in vivo*	Chu et al. (2021)
EpCAM	ep133	EHLHCLGSLCWP	No	Yes	NR	EpCAM + tumors, *in vitro*	Park et al. (2021)

NR, not reported.

### 4.7 Routes of administration for pulmonary delivery

For pulmonary EV therapy, the main routes of administration explored to date are systemic intravenous infusion and local respiratory delivery, including nebulized inhalation, direct intratracheal instillation, and, in some preclinical studies, intranasal administration. Among the local delivery approaches, nebulized inhalation currently has the strongest translational appeal, as it is noninvasive, has already entered clinical testing, and is designed to enhance airway and alveolar exposure while limiting the liver- and spleen-dominant systemic distribution typically observed after intravenous administration. Published clinical experience remains limited, but the available evidence includes two pilot studies of nebulized MSC-derived exosomes in COVID-19 and a randomized, single-blind, placebo-controlled phase I trial of nebulized hUCMSC-EVs in pulmonary fibrosis. Collectively, these studies support feasibility and short-term safety, while providing only preliminary indications of clinical benefit rather than conclusive evidence of efficacy (Chu et al., 2022; Zhu et al., 2022; Tolomeo et al., 2023; Li et al., 2025).

Intravenous administration remains the most established systemic route and has also been evaluated in randomized clinical testing for COVID-19-associated respiratory failure/ARDS. However, its biodistribution is inherently less lung-selective and therefore better aligned with applications requiring systemic immunomodulation rather than direct epithelial targeting (Lightner et al., 2023). Preclinical biodistribution studies support this distinction: following intravenous injection, EVs accumulate predominantly in the liver and spleen, whereas intratracheal administration results in substantially greater lung localization. Intranasal delivery is attractive because it is simple and noninvasive, but in mice it directs a greater proportion of EV signal toward the brain rather than the distal lung, making it a less reliable route for lung-targeted delivery in this setting (Tolomeo et al., 2023).

These biodistribution findings are supported by preclinical efficacy studies. In murine acute lung injury, inhaled EVs showed greater therapeutic benefit than tail-vein administration, while in experimental bronchopulmonary dysplasia, intratracheal delivery of MSC-EVs likewise attenuated lung injury. However, intratracheal instillation remains primarily a preclinical experimental benchmark for local delivery, as it is invasive and does not fully reproduce clinical aerosol delivery (Porzionato et al., 2021; Zhao et al., 2022). Taken together, current evidence supports nebulized inhalation as the most attractive route for EVs intended to act within the lung epithelium, whereas intratracheal instillation is best viewed as an experimental local-delivery benchmark and intravenous infusion as a clinically feasible but less selective alternative.

Preclinical efficacy studies are broadly consistent with the observed biodistribution patterns. In murine acute lung injury, inhaled EVs were more effective than tail-vein administration, whereas in experimental bronchopulmonary dysplasia, intratracheally delivered MSC-EVs likewise mitigated lung injury (Zhao et al., 2022; Porzionato et al., 2021). Although intratracheal instillation is useful for demonstrating the therapeutic potential of local EV delivery in preclinical studies, it remains primarily an experimental approach, as it is invasive and less physiological than inhalation (Driscoll et al., 2000; Kumar Subramani et al., 2024). Overall, the available evidence supports nebulized inhalation as the most promising local route for EVs intended to act within the lung epithelium, whereas intratracheal instillation remains mainly an experimental approach to local delivery and intravenous administration a clinically feasible but less lung-selective alternative.

## 5 Conclusion

Airway basal cells represent a compelling target for durable genetic intervention in cystic fibrosis, as correction of this stem/progenitor compartment could, in principle, support long-term restoration of function across regenerated epithelial lineages. However, this strategy also poses a substantial delivery challenge, because basal cells are anatomically shielded within the pseudostratified airway epithelium and no single surface marker currently combines ideal specificity, accessibility, and functional relevance for selective *in vivo* targeting. The available evidence instead suggests that successful basal cell targeting will likely require a combination of approaches, including careful marker prioritization, context-aware ligand design, and delivery platforms capable of penetrating diseased airway barriers. In this regard, EVs and vesicle-mimetic systems are particularly attractive because of their versatility, engineering potential, and compatibility with complex nucleic acid and protein cargoes, although major challenges remain in improving airway penetration, controlling tropism, and achieving efficient and reproducible delivery to the basal cell compartment. While this Review focuses on cystic fibrosis, the same conceptual framework may also be relevant to other inherited airway diseases, including primary ciliary dyskinesia, in which durable correction of airway progenitors could likewise support sustained replenishment of functional multiciliated cells. Continued progress in airway cell targeting, vesicle engineering, and disease-relevant *in vivo* models will therefore be essential for translating basal cell-directed therapies from a compelling concept into a practical therapeutic strategy.
